# The 5^th^ edition of WHO classification of tumors of endocrine organs: changes in the diagnosis of follicular-derived thyroid carcinoma

**DOI:** 10.1007/s12020-023-03336-4

**Published:** 2023-03-25

**Authors:** Fulvio Basolo, Elisabetta Macerola, Anello Marcello Poma, Liborio Torregrossa

**Affiliations:** Department of Surgical, Medical, Molecular Pathology and Critical Area - University of Pisa, via Savi 10, Pisa, 56126 Italy

**Keywords:** Endocrine pathology, Follicular-derived thyroid cancer, WHO classification system, Diagnostic pathology

## Abstract

The 5^th^ edition of the World Health Organization (WHO) classification of endocrine tumors was released in 2022. Several novelties have been introduced concerning the nomenclature and histopathological diagnosis of follicular-derived thyroid neoplasms. Tumor types have been sharply classified according to prognostic risk categories into benign tumors, low-risk neoplasms and malignant neoplasms. A grading system for differentiated thyroid carcinomas has been implemented with the aim of improving the stratification of tumors. Particular attention has been paid to the molecular profile of well-differentiated histotypes. In this review, the main changes introduced by the latest edition of the WHO system are presented. The practical effects on the diagnostic pathology of thyroid tumors, along with the clinical implications expected with the new classification scheme, are critically discussed.

## Introduction

**Fig. 1:**
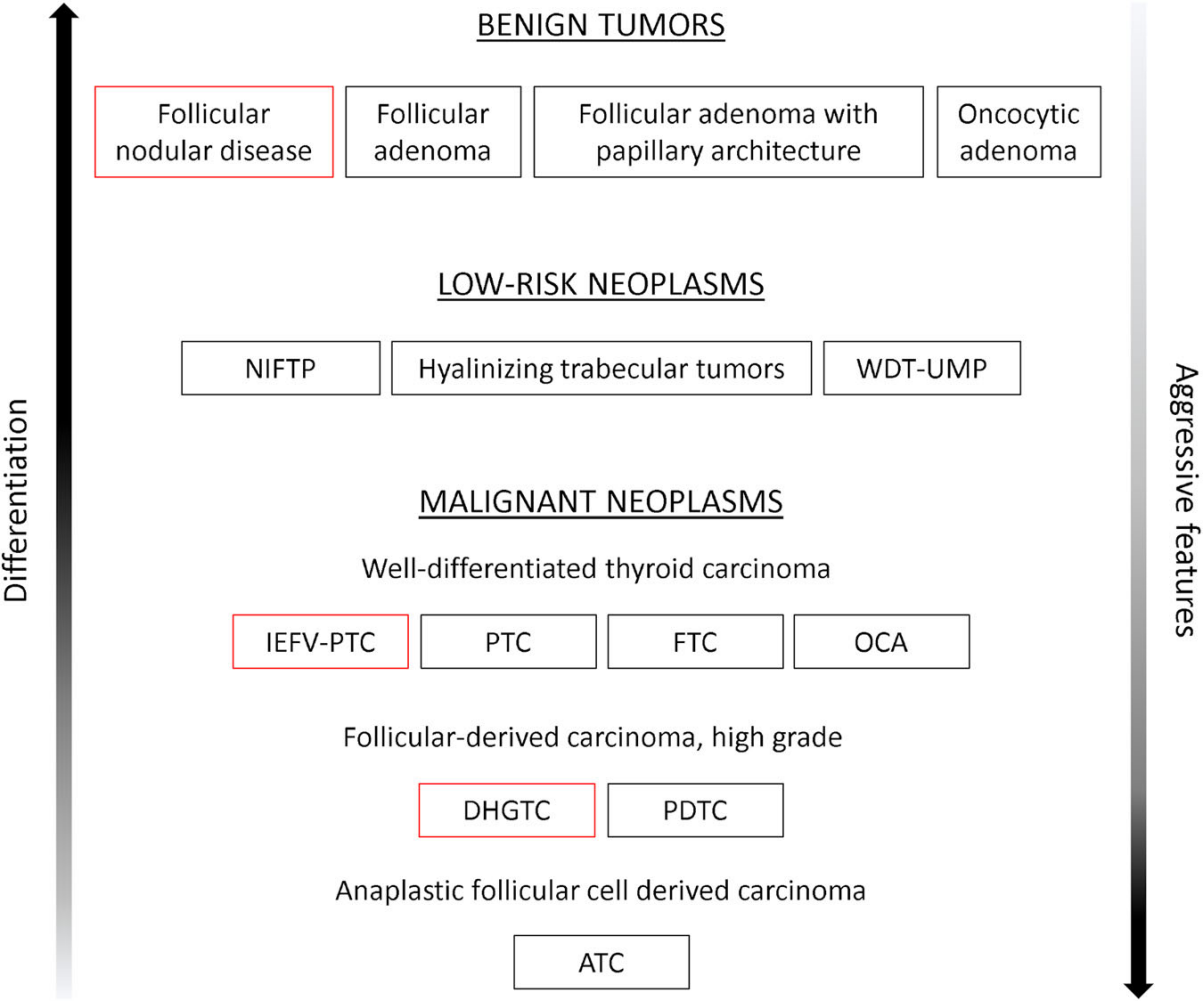
The World Health Organization classification of follicular cell-derived thyroid neoplasms, 5^th^ edition. Thyroid tumors have been subdivided into benign tumors, low-risk neoplasms and malignant neoplasms. Red squares indicate the newly introduced subtypes. Abbreviations: NIFTP noninvasive follicular thyroid neoplasm with papillary-like nuclear features, WDT-UMP well-differentiated tumor of uncertain malignant potential, IEFV-PTC invasive encapsulated follicular variant papillary thyroid carcinoma, PTC papillary thyroid carcinoma, FTC follicular thyroid carcinoma, OCA oncocytic thyroid carcinoma, DHGTC differentiated high-grade thyroid carcinoma, PDTC poorly differentiated thyroid carcinoma, ATC anaplastic thyroid carcinoma

The 4^th^ edition of the WHO classification of tumors of endocrine organs was published in 2017 [1]. After only 5 years, a re-edited version of this important reference guide for pathologists and clinicians came to light [2]. Follicular-derived thyroid tumors were categorized into “Benign tumors”, “Low-risk neoplasms” and “Malignant neoplasms” (Fig. 1); this clear-cut subdivision appears far more linear than the previous one, where a list of histotypes rather than a real categorization was present. The new version seems to give more importance to the integration of morphological and molecular characteristics of tumors. In this brief review, the main novelties of the 5^th^ edition of the WHO classification of follicular-derived thyroid tumors are summarized, discussing the expected consequences for pathologists and clinicians in real-world practice.

## Benign tumors

In the 5^th^ edition of the WHO classification, more space is given to the group of benign thyroid lesions. The follicular adenoma, an encapsulated tumor with follicular architecture characterized by clonal expansion and *RAS*-like alterations, was already present in the previous edition. The novelty is represented by a more detailed definition of the well-known multinodular goiter. Multinodular goiter is a clinical condition characterized by multiple, non-malignant thyroid nodules originated by the proliferation of follicular cells. So far, the histological appearance of these lesions has determined various definitions: micro- and/or macro-follicular nodules, hyperplastic nodules, colloidal nodules, and adenomatous nodules. However, the nature of these nodules can be both hyperplastic and neoplastic, as demonstrated by the occurrence of clonal molecular alterations. To uniform the terminology, the definition of *thyroid follicular nodular disease* has been introduced in the new WHO classification scheme [2].

Two other entities have been included in the category of benign tumors, namely the follicular adenoma with papillary architecture and the oncocytic adenoma. These neoplasms were already present in the 4^th^ edition of the WHO and their histopathological characteristics have not been subject to significant modifications. The follicular adenoma with papillary architecture was previously mentioned as one of the follicular adenoma variants. It shows papillary structures but lacks papillary-like nuclear features.

The oncocytic adenoma, a noninvasive encapsulated neoplasm composed by >75% oncocytic cells, was formerly defined as “Hürthle cell adenoma” and included into the section of the “Hürthle cell tumors”. In the new WHO edition, it is recommended that these neoplasms should be referred to as oncocytic tumors rather than Hürthle cell tumors [2].

## Low-risk neoplasms

The 5^th^ WHO classification scheme has introduced the class of low-risk neoplasms, which include noninvasive follicular neoplasms with papillary-like nuclear features (NIFTP), tumors of uncertain malignant potential and hyalinizing trabecular tumors. This category was thought to be able to bridge the gap between benign neoplasms, such as nodules in the context of follicular nodular disease or follicular adenoma, and frankly malignant tumors [3]. Low-risk neoplasms are characterized by an excellent prognosis but cannot be categorized as benign. In the previous edition of the WHO classification, NIFTP and tumors with uncertain malignant potential were referred to as “Other encapsulated follicular-patterned tumors”, while hyalinizing trabecular tumors were placed separately. Regarding NIFTPs, the criterion of less than 1% of papillae has been maintained although some authors have proposed that NIFTP should have a total absence of papillae [4]. Tumors sized ≤1 cm fulfilling the specific diagnostic criteria should be diagnosed as NIFTP.

## Malignant neoplasms

### Papillary thyroid carcinoma

The term “variant” in the papillary thyroid carcinoma (PTC) types has been formally replaced with “subtype”. The PTC subtypes in the 2022 WHO classification have remained unchanged, and the diagnostic criteria substantially maintained, with the following exceptions:

- the invasive encapsulated follicular variant PTC is no longer included among PTCs, as extensively discussed in the next paragraph.
- the tall cell subtype is defined by the presence of ≥30% of tumor cells with tall cell features; according to the new WHO indications, the tall cells are at least three times taller than wide, while the criterion was previously two to three times taller than wide.
- the diagnosis of solid PTC subtype now requires that >50% of tumor shows a solid, trabecular or nested growth pattern; in the WHO 4^th^ edition, it was established that all or nearly all the tumor should be composed by solid, trabecular, or nested areas.
- in the WHO 4^th^ edition, it was clearly stated that the hobnail variant PTC should be defined by the presence of at least 30% of cells with hobnail features; in the WHO 5^th^ edition, this cutoff is not explicitly mentioned.
- PTCs sized ≤1 cm (microPTC) are not considered as a unique PTC subtype. MicroPTCs generally show an indolent behavior. However, they rarely progress and can cause recurrence; therefore, a classification based on their histo-morphology rather than mere tumor size has been considered more appropriate [3].
- the cribriform morular tumor is no longer a PTC subtype; it has been placed among the “thyroid tumors of uncertain histogenesis”.

### Invasive encapsulated follicular variant PTC *versus* infiltrative follicular PTC subtype

**Fig. 2:**
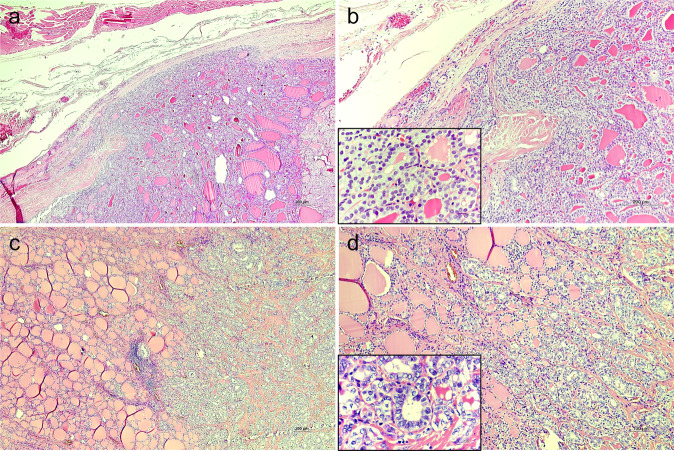
Representative histopathologic images of invasive encapsulated follicular variant PTC (**a**, **b**) and infiltrative follicular subtype of PTC (**c**, **d**) (haematoxylin/eosin staining). **a**, **b** The minimally invasive encapsulated follicular variant PTC shows focal invasion of the neoplastic capsule with a mushroom aspect (original magnification ×4 and ×10, respectively). In the insect, the neoplastic cells show mild nuclear atypia (original magnification ×60). **c**, **d** The infiltrative follicular subtype of PTC exhibits a pattern of growth towards the adjacent thyroid parenchyma without evidence of neoplastic capsule (original magnification ×4 and ×10, respectively). In the insect, the nuclear atypia of the conventional PTC is more evident than that observed in the invasive encapsulated follicular variant PTC (original magnification ×60)

Over the last years, the diagnosis of the follicular variant PTC has been subjected to a series of modifications. The noninvasive encapsulated type has been reclassified as NIFTP, which was already present in the previous WHO classification system [5]. In the WHO 5^th^ edition, follicular variant PTCs have been further subdivided into “encapsulated with invasion” and “infiltrative” subtypes. The invasive encapsulated follicular variant PTC is not included in the PTC category and is considered as a separate entity among malignant neoplasms; the infiltrative follicular variant is still considered a PTC subtype. The infiltrative type is in fact very similar to the conventional PTC in terms of infiltrative growth pattern, but it shows almost exclusively a follicular architecture (Fig. 2).

**Fig. 3:**
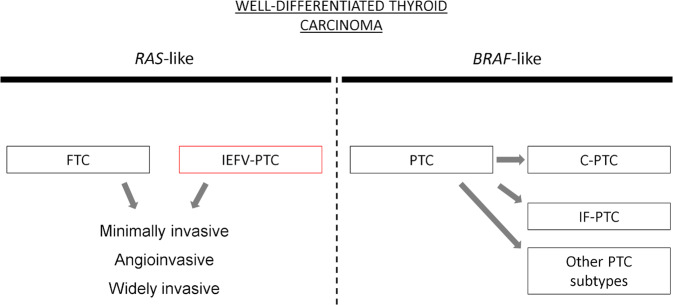
The scheme shows how well-differentiated thyroid carcinomas can be separated according to their molecular landscape. As a rule, papillary thyroid carcinomas (PTCs) are *BRAF*-like neoplasms that encompass the classical type (C-PTC) and other subtypes, including also the infiltrative follicular subtype (IF-PTC). *RAS*-like lesions include the invasive encapsulated follicular variant PTC (IEFV-PTC), highlighted by a red square, and the follicular thyroid carcinoma (FTC). Oncocytic thyroid carcinomas, although belonging to well-differentiated thyroid carcinomas, have not been included in the scheme because they do not show a *BRAF*-like nor a *RAS*-like molecular profile. Abbreviations: FTC follicular thyroid carcinoma, IEFV-PTC invasive encapsulated follicular variant papillary thyroid carcinoma, PTC papillary thyroid carcinoma, C-PTC classic papillary thyroid carcinoma, IF-PTC infiltrative follicular papillary thyroid carcinoma subtype

Like the follicular thyroid carcinoma (FTC), the invasive encapsulated follicular variant PTC should be subdivided into minimally invasive, angioinvasive and widely invasive type. It is interesting to highlight that the separation of encapsulated invasive follicular variants from PTCs has been essentially guided by molecular reasons. Indeed, it is known that both noninvasive and invasive encapsulated follicular variant PTCs typically show *RAS*-like molecular alterations, while infiltrative follicular PTC subtypes display a *BRAF*-like phenotype, similarly to classic PTCs [6]. This fact indicates that the key features of PTCs no longer lie in the typical nuclear changes (also present in NIFTP and in the invasive encapsulated follicular variant PTC), but rather in the tumor molecular features, and PTC is substantially a *BRAF*-like carcinoma (Fig. 3).

The distinction of the former follicular variant PTC into invasive encapsulated and infiltrative forms seems to reflect the intent of further sharpening the risk stratification of these lesions. In general, invasive encapsulated follicular variant PTCs are known to have a better prognosis than infiltrative PTCs [7]. Clinically, the European and American Thyroid Association guidelines define as low risk all the non-aggressive and intrathyroidal PTC subtypes with no loco-regional involvement [8, 9]. A sharper distinction of the follicular variant PTC at the histological level is likely to have no consequences on the management of these patients. However, the separation of invasive encapsulated follicular variants from PTCs implicates that pathologists should perform a careful examination of the tumor capsule and accurately describe the degree of tumor invasiveness in the histological report. This is particularly important since, as in the case of widely invasive follicular carcinoma, patients with widely invasive encapsulated follicular variants are likely to be at higher risk of developing distant metastases and could benefit from a more aggressive treatment [2]. Whether widely invasive encapsulated follicular variant PTCs should be considered in the same way as the other aggressive PTC and FTC subtypes will probably be a controversial issue in the next future, until more prospective data are available.

### Oncocytic thyroid carcinoma

The “Hürthle cell carcinoma” is called “oncocytic thyroid carcinoma” (OCA) in the 2022 WHO classification [2]. Besides the terminology, the histo-pathological criteria for diagnosis of OCAs and their subclassification into minimally invasive, angioinvasive, and widely invasive type have remained unchanged. OCAs are composed by more than 75% cells showing the typical appearance of oncocytes, that is large size, deeply eosinophilic and granular cytoplasm, and large nuclei with prominent nucleoli.

### High-grade follicular-derived thyroid carcinoma

In the 2022 WHO classification a new class of malignant tumors, namely the high-grade follicular-derived thyroid carcinoma, has been introduced: [2]. This class includes tumors with a prognosis that is halfway between well-differentiated tumors and anaplastic carcinoma, and it encompasses differentiated high-grade thyroid carcinoma and poorly differentiated thyroid carcinoma. For the latter, the diagnostic criteria have not been modified, while the novelty is represented by the differentiated high-grade thyroid carcinoma. To describe the characteristics of this new histo-pathological entity, it is necessary to recall the diagnostic criteria for poorly differentiated carcinoma. According to the Turin consensus, adopted also by the WHO Committee, poorly differentiated carcinoma shows partial loss of its cytological and architectural differentiation, with no papillary-like nuclear changes. Moreover, it shows a solid, trabecular or insular growth pattern and at least one of the following characteristics: convoluted nuclei, ≥3 mitosis per 10 high-power fields (HPF), and tumor necrosis [1]. The Memorial Sloan Kettering Cancer Center (MSKCC) has adopted an alternative definition of poorly differentiated thyroid carcinoma, for which follicular cell differentiation and papillary nuclear features are also allowed [10]. Therefore, the MSKCC tumors include poorly differentiated tumors as defined by the Turin proposal, and also well-differentiated carcinomas with a high mitotic index (≥5 mitosis per 10 HPF) and/or tumor necrosis. On the contrary, only part of the MSKCC carcinomas also fulfill the diagnostic criteria of the Turin consensus (35–65% of cases) [11].

**Fig. 4:**
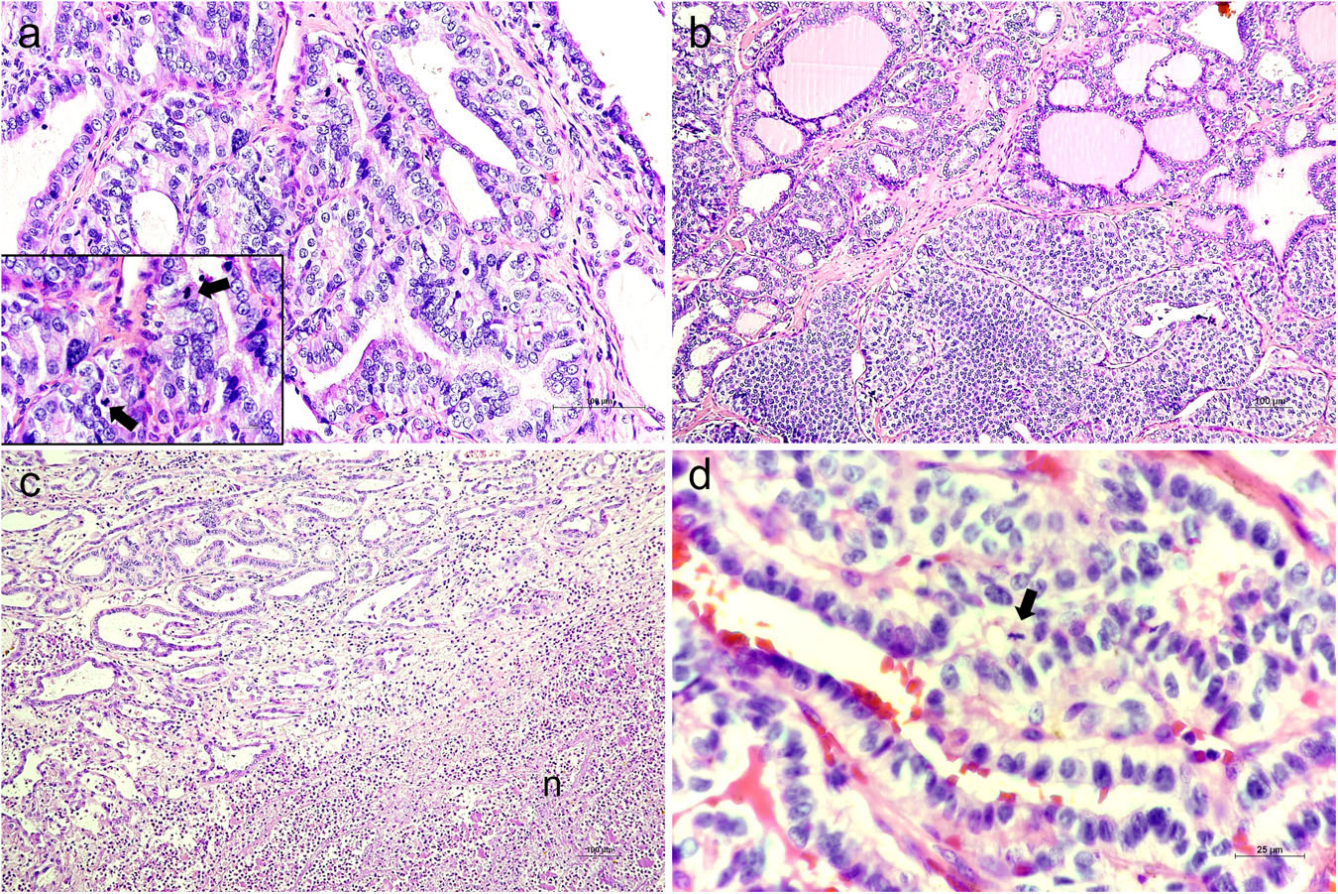
Histopathological pictures of high-grade follicular-derived thyroid carcinoma cases (haematoxylin/eosin staining). **a** High-grade follicular subtype PTC (original magnification ×20); in the insect, arrows indicate mitotic figures at higher magnification (x60). **b** Picture showing a differentiated high-grade follicular subtype PTC (upper left corner) and poorly differentiated (insular and solid growth patterns) component (original magnification ×10). **c** Differentiated high-grade carcinoma showing follicular subtype PTC in association with wide areas of tumor necrosis (original magnification ×10; “n” indicates necrosis). **d** High-grade tall cell PTC subtype (original magnification ×60; arrow indicates a mitotic figure)

From a molecular point of view, poorly differentiated carcinomas defined by the Turin consensus harbor more frequently *RAS* mutations, while MSKCC cases are enriched with *BRAF* mutations [12]; 40–55% of tumors harbor *TERT* promoter mutations [12, 13]. Several authors have tried to compare the clinical outcome of Turin- and MSKCC-diagnosed poorly differentiated carcinomas. The MSKCC criteria identify a larger group of tumors but provide similar prognostic information compared to the Turin criteria [14, 15]. This evidence confirms the important role of high mitotic rate and tumor necrosis as predictors of poor prognosis independently of tumor dedifferentiation, as also acknowledged in the 2017 WHO book [1, 16]. In a study conducted by Xu and colleagues in 2021, the clinico-pathological features of 164 high-grade carcinomas not fulfilling the Turin criteria and 200 Turin-defined poorly differentiated carcinomas were compared [13]. Besides the confirmation that Turin carcinomas show a higher prevalence of *RAS* mutations and are more prone to spread distantly, the authors found that disease-specific survival and locoregional recurrence-free survival rates did not significantly differ between the two groups. The survival rates observed in the entire cohort indicated an overall poor prognosis: 5-year overall survival (OS) 75%; 10-year OS 54%; and 20-year OS 28%, compared to the survival rates of differentiated thyroid cancer (10-year OS > 90%) [17]. Therefore, the presence of high-grade features in well-differentiated histotypes allows to capture a class of intermediate/high-risk tumors that would be otherwise diagnosed as PTC, FTC, or OCA. In the WHO 5^th^ edition, the differentiated high-grade thyroid carcinoma has been introduced (Fig. 4), and this definition includes any differentiated thyroid carcinoma showing ≥5 mitosis per 2 mm^2^ and/or tumor necrosis. It is worth noting that the conventional way of assessing the mitotic activity was to count the number of mitoses in 10 HPF. In the new WHO indications, to allow a better standardization of this measure it is recommended that mitoses should be counted in a 2 mm^2^ spot, which is approximately equivalent to 10 HPF [18].

It is expected that a subgroup of differentiated thyroid cancers, especially among aggressive subtypes such as tall cell and hobnail PTCs, widely invasive FTCs, and widely invasive OCAs, will fulfill the diagnostic criteria for differentiated high-grade thyroid carcinomas [3]. In rare cases, totally encapsulated tumors fulfilling diagnostic criteria for NIFTP can show ≥5 mitosis per 2 mm^2^ and/or tumor necrosis; a diagnosis of differentiated high-grade follicular variant PTC should be made [2]. The incidence of high-grade differentiated thyroid carcinoma must be established; however, it should reflect the prevalence of MSKCC carcinomas, excluding the portion of the Turin poorly differentiated cancers.

When a tumor shows high-grade features and a mixture of differentiated and poorly differentiated areas (Fig. 4b), the least differentiated tumor component, even if non-predominant, should be recorded; in the pathological report all the tumor components and their pathological characteristics should be described [2, 3].

### Squamous cell carcinoma

Anaplastic carcinoma of the thyroid is the most aggressive thyroid malignancy [19]. It is characterized by the loss of differentiation, even if differentiated tumor areas can be present. Anaplastic carcinoma mainly shows epithelioid and spindle cell areas. In the previous edition of WHO, squamous cell carcinoma had an independent section, while it is now rated as a subtype of anaplastic thyroid cancer. Squamous cell carcinoma shows evident squamous differentiation and can be associated with PTC, typically of high grade [20]. The follicular origin of the tumor can be documented by the immunohistochemical expression of TTF-1 and PAX-8 [2, 18]. In general, the prognosis of squamous cell carcinoma appears similar to that of anaplastic carcinoma, justifying the assimilation of this tumor as one of its morphological subtypes [3]. Squamous cell carcinoma often harbors *BRAF* mutations, and this is of particular importance, since patients with *BRAF*-mutant anaplastic thyroid carcinoma can benefit from treatment with targeted drugs [19].

## What changes for pathologists

In the 5^th^ edition of the WHO classification of thyroid tumors, several novelties essentially concern terminological issues, i.e., the follicular nodular disease, and the PTC “subtypes” instead of “variants”. These changes do not reflect modifications in the diagnostic criteria, and they are not expected to have clinical consequences. In contrast, in the new edition, there is the necessity to provide a more accurate histological characterization of tumors, especially differentiated carcinomas.

Paramount hallmarks of histological diagnosis of both poorly differentiated and differentiated high-grade thyroid carcinoma are the high mitotic index and the identification of tumor necrosis. In general, tumor necrosis can appear as comedonecrosis-like small foci or as large areas of ghost neoplastic cells floating in necrotic material. During the histopathological examination, the discrimination between real tumor necrosis and necrotic hemorrhagic events, such as those associated with fine-needle aspiration procedures, is crucial [21]. Moreover, a complete examination of the sample should be stressed to identify even small foci of tumor necrosis.

As regards the mitotic count, in the previous edition of WHO, the presence of solid, trabecular or insular growth associated with at least 3 mitoses per 10 HPF was sufficient to label a carcinoma as poorly differentiated, also in the absence of tumor necrosis. In the new WHO classification, the pathologist is called to identify and eventually count the mitoses even in presence of well-differentiated thyroid carcinomas, in order to eventually diagnose them as differentiated high-grade carcinomas. Therefore, the microscopic examination should be more accurate and exhaustive in all the thyroid tumor cases, eventually coupled with immunohistochemical staining for the evaluation of proliferative activity, such as Ki-67 [18].

Another challenging aspect of the new edition of WHO is the morphological distinction between the invasive encapsulated follicular variant PTC and the infiltrative follicular PTC subtype. In presence of a clear neoplastic capsule surrounding the tumor, identification of the invasive encapsulated follicular variant PTC should be easy. The tumor should then be categorized as minimally invasive, angioinvasive or widely invasive. In the case of a follicular variant PTC showing a massive invasion towards the tumor capsule, which might not be evident or only residual, the distinction between a widely invasive encapsulated follicular variant PTC and an infiltrative follicular subtype could be difficult, and is likely to be more subjective. In these specific circumstances molecular characterization could be useful to discriminate between the two entities: in most cases the infiltrative follicular subtype is a *BRAF*-like tumor, while the invasive encapsulated subtype is a *RAS*-like tumor [2].

## Conclusions

In conclusion, the 5^th^ edition of the WHO classification of follicular-derived thyroid tumors introduced several novelties, some of which requiring a more accurate histological and molecular characterization of tumors. The introduction of histopathological tumor grading is likely to improve the risk stratification of patients. However, the proportion of PTC, FTC and OCA that will eventually be diagnosed as differentiated high-grade thyroid tumors is yet to be established. The overall clinical impact of the new classification scheme will be monitored in the next years.
